# Enhancing Human Post‐Exposure Rabies Prophylaxis in Canine Rabies Endemic Regions With an Interactive Web Solution

**DOI:** 10.1002/puh2.70030

**Published:** 2025-02-07

**Authors:** Aniruddha V. Belsare, Deborah J. Briggs, Gyanendra Gongal, Reeta S. Mani, Charles E. Rupprecht

**Affiliations:** ^1^ Auburn University College of Forestry, Wildlife and Environment Auburn Alabama USA; ^2^ Auburn University College of Veterinary Medicine Auburn Alabama USA; ^3^ Kansas State University College of Veterinary Medicine Manhattan Kansas USA; ^4^ World Health Organization, WHO Regional Office for South–East Asia New Delhi India; ^5^ Department of Neurovirology National Institute of Mental Health and Neurosciences Bengaluru Karnataka India; ^6^ LYSSA LLC Lawrenceville Georgia USA

**Keywords:** dog, prophylaxis, rabies, shiny app, zoonosis

## Abstract

Despite the availability of effective biologics, rabies continues to impact several low‐ and middle‐income countries. More than 50,000 people succumb to rabies every year due to the lack of timely and appropriate post‐bite care. Biologics used in post‐exposure prophylaxis, such as rabies vaccine, rabies immune globulins, and rabies monoclonal antibodies, are not readily available and often in short supply in the developing world, especially in Asia and Africa, where rabies is endemic in domestic dog populations and poorly controlled. Moreover, many healthcare professionals in these settings have less than ideal knowledge of current post‐exposure prophylaxis guidelines. To combat this, we have integrated current post‐bite prophylaxis guidelines and accurate information about the availability of rabies biologics into an interactive, user‐friendly web application, ZeroRabiesApp (ZRA). Designed as a point‐of‐care tool for treating dog bite cases, ZRA generates a customized post‐exposure prophylaxis schedule using the user‐provided date of exposure. The schedule is based on the latest guidelines developed by the World Health Organization (WHO) and the Centers for Disease Control and Prevention (CDC). The ZRA also provides access to the rabies biologics database that can be used to find the nearest locations where rabies biologics needed for post‐exposure prophylaxis are currently available. The freely available app can offer a simple, real‐time, and local solution to prevent human rabies deaths following potentially rabid exposures like dog bites.

## Graphical abstract

**ZeroRabiesApp (ZRA)** is an interactive web application designed to ensure prompt and appropriate post‐exposure treatment for dog bite victims. ZRA enhances compliance with post‐exposure prophylaxis (PEP) by offering a user‐friendly, point‐of‐care tool aligned with the latest guidelines. It also provides access to a rabies biologics database, helping users locate nearby facilities with PEP products in stock for timely treatment.

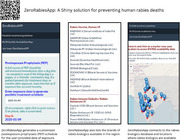

## 1 Background

Rabies is a neglected disease, disproportionately affecting impoverished communities in Africa and Asia, despite the availability of effective vaccines for both animals and humans for over a century. Domestic dogs are the primary reservoirs of the rabies virus in these regions. Unfortunately, concerted efforts to control rabies among dog populations have been lacking in many countries in Asia and Africa. As a result, each year, tens of thousands of human lives in low‐ and middle‐income countries (LMICs) are lost to dog‐mediated rabies. India, in particular, grapples with an alarming estimate of 17.4 million dog bite cases and 20,000 dog‐mediated human rabies deaths annually [1].

Human rabies prevention in India has encountered significant hurdles, primarily stemming from limited knowledge about proper post‐exposure prophylaxis (PEP) and inconsistent access to rabies biologics [2, 3]. Regrettably, the lack of readily available guidance on the clinical management of human rabies condemns individuals to endure excruciating suffering, ultimately culminating in a tragic and inevitable loss of life [4]. The prompt administration of appropriate PEP following a dog bite is crucial to prevent rabies [5]. However, many healthcare workers in LMICs often lack the necessary knowledge about proper PEP [2]. Additionally, rabies biologics, such as the rabies vaccine (RV), rabies immune globulin (RIG), and rabies human monoclonal antibodies (mAb) used in PEP, are frequently scarce in developing regions, particularly in Africa and Asia [6, 7]. Typically, dog bite patients or their caregivers, often residing in remote rural areas, must embark on arduous journeys to acquire these biologics for PEP, resulting in delays or even the inability to receive treatment. In addition to promoting public awareness about dog bite prevention and proper medical care after such incidents, there is a pressing need for a mechanism to track the real‐time availability of PEP products.

## 2 A Shiny Solution

**FIGURE 1:**
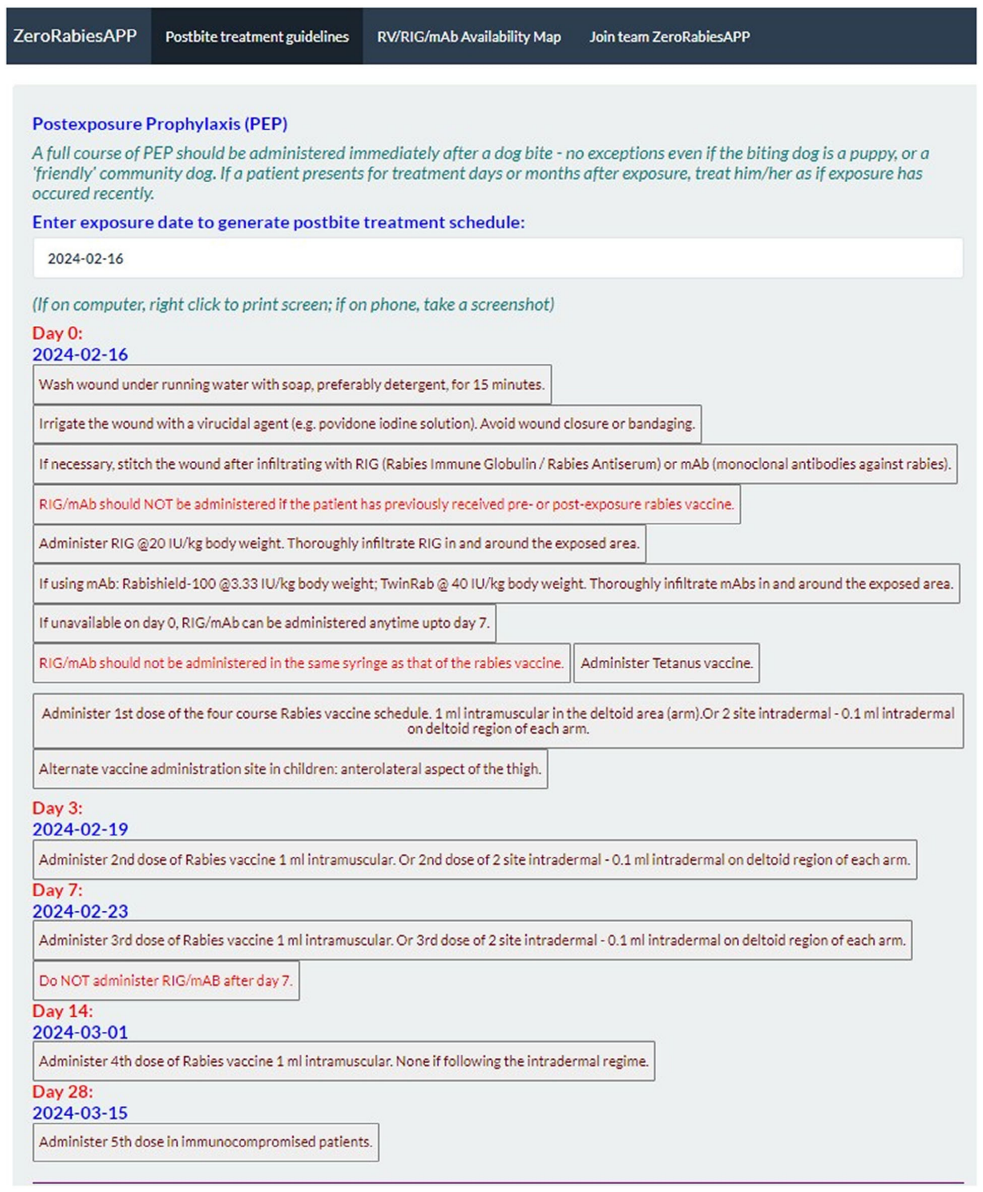
Screenshot of the ZeroRabiesAPP (ZRA) illustrating the customized post‐exposure prophylaxis (PEP) schedule generated for an exposure date entered by the user. mAb, monoclonal antibody; RIG, rabies immune globulin; RV, rabies vaccine.

To address these challenges, we have created an online web application called ZeroRabiesAPP (ZRA), utilizing the Shiny package in R software. ZRA has been designed as an interactive point‐of‐care tool for bite management, granting users access to the latest guidelines developed by the World Health Organization (WHO) (https://www.who.int/news‐room/fact‐sheets/detail/rabies) and the US Advisory Committee on Immunization Practices https://www.cdc.gov/rabies/hcp/prevention‐recommendations/post‐exposure‐prophylaxis.html. Furthermore, the ZRA generates a customized PEP schedule based on the user‐provided bite date (day of exposure), as illustrated in Figure 1. The customized PEP schedule can be saved as a snapshot or printed and shared with a healthcare provider. The PEP schedule generated by the ZRA eliminates any uncertainty regarding the timing of rabies biologics administration.

**FIGURE 2:**
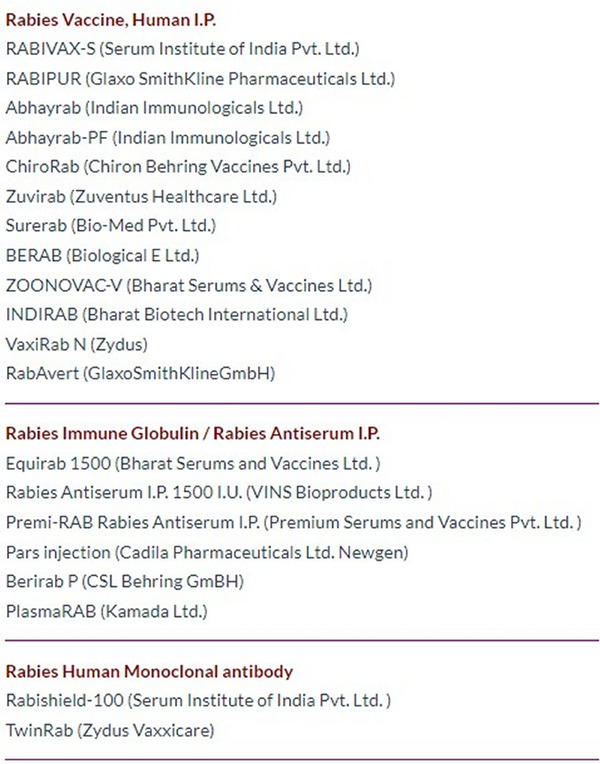
List of rabies vaccine (RV), rabies immune globulin (RIG), and rabies human monoclonal antibodies (mAbs) brands available in India.

Along with the customized PEP schedule, the ZRA provides a list of RV, RIG, and mAb brands available in the region (Figure 2). The ZRA also offers access to the rabies biologics database, which can be used to locate the nearest places where PEP products are currently in stock, ensuring timely access to necessary treatment (Tab: RV/RIG/mAb Availability Map). The rabies biologics database is a comprehensive compilation of facilities in India (pharmacies, clinics, hospitals, etc.) where rabies biologics are presently available.

**FIGURE 3:**
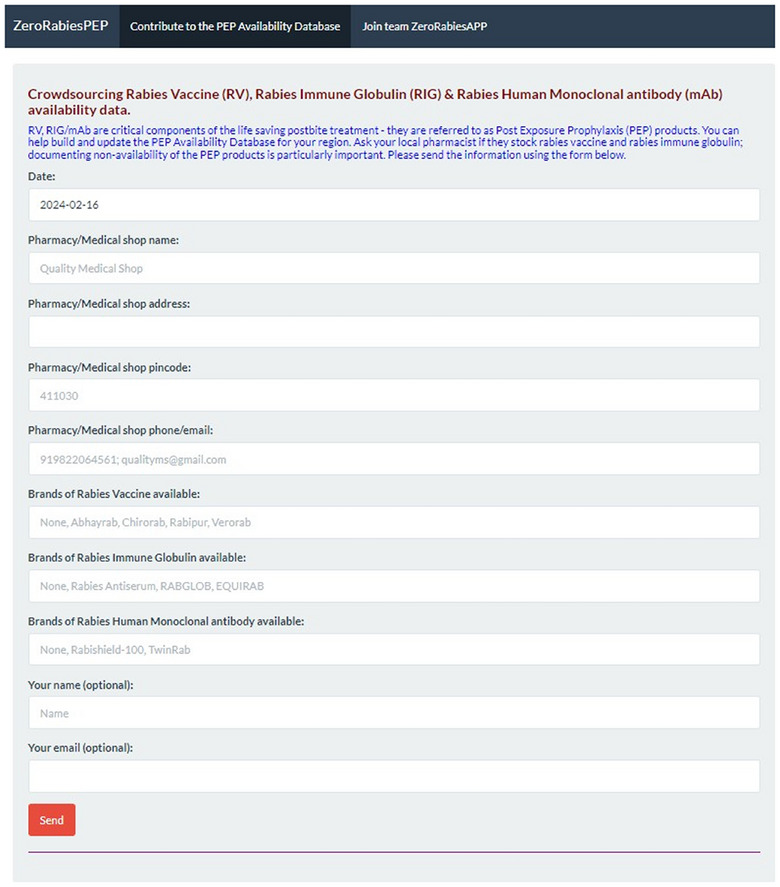
The ZeroRabiesPEP (ZRP) is designed to facilitate citizen involvement in the process of developing rabies biologics databases. PEP, post‐exposure prophylaxis.

The rabies biologics database is compiled and updated using another online web application, ZeroRabiesPEP (ZRP). ZRP is integrated with the ZRA and serves as a crowdsourcing tool, facilitating easy submission of PEP availability information (Figure 3). The ZRA team (Tab: Join team ZRA) comprises volunteers facilitating the compilation of a comprehensive database of facilities in India (pharmacies, clinics, hospitals, etc.) where rabies biologics are currently available. The volunteers carefully review the information provided by citizens through ZRP before it is integrated into the rabies biologics database. This includes calling the facilities to ensure the accuracy of the information submitted through ZRP. Ethics approval was not required as no human data or samples were collected for this work.

**FIGURE 4:**
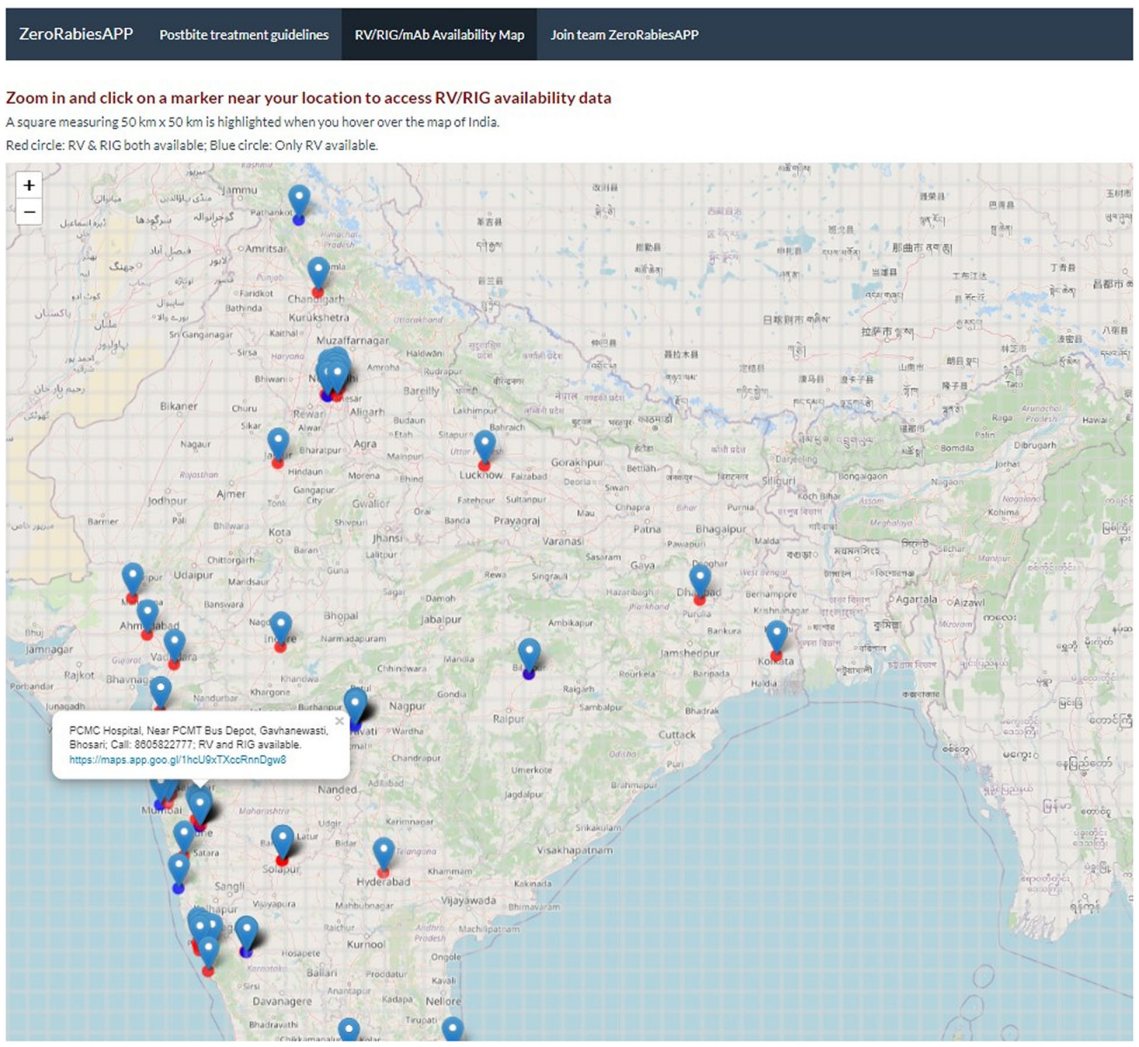
The RV/RIG/mAb Availability Map in the ZeroRabiesAPP (ZRA) is connected with a rabies biologics database. Red circles indicate availability of rabies vaccine (RV), rabies immune globulin (RIG), and/or rabies human monoclonal antibodies (mAb) whereas blue circles indicate availability of rabies vaccine only. The user can click on a marker to access information as illustrated.

The RV/RIG/mAb Availability Map in ZRA is dynamically connected to the rabies biologics database and shows the locations where rabies biologics are available as a marker on the map. Locations with RV, RIG, and/or mAb are marked with red circles, whereas places with only RV are denoted by blue circles. The user can click on a marker to access contact details of the pharmacy/healthcare facility (Figure 4). Access to the rabies biologics database can markedly reduce the time and energy required to locate these biologics following a potential exposure. Furthermore, the RV/RIG/mAb availability map is superimposed with a grid consisting of 50 km × 50 km patches, as depicted in Figure 4.

Our target is to ensure that there is at least one marker indicating the availability of these products within each square patch on the RV/RIG/mAb availability map in the ZRA. The ZRA is freely available at: https://anyadoc.shinyapps.io/ZeroRabiesINDIA/ and the ZRP is freely available at: https://anyadoc.shinyapps.io/ZeroRabiesPEP/.

## 3 Discussion

The overarching goal of the ZRA is to support the global strategic plan to end human deaths from dog‐mediated rabies by 2030 (Zero by 30; https://iris.who.int/handle/10665/272756). The ZRA addresses the problem of poor PEP compliance by creating a freely accessible rabies biologics database. This initiative also serves to identify regions where dog bite victims might have to travel considerable distances to obtain PEP products. Furthermore, the rabies biologics database can be employed to formulate strategies tailored to local needs aimed at enhancing the affordability and accessibility of rabies PEP, such as exploring options for intradermal vaccination.

These apps are designed to be customizable and adaptable to different regions where canine rabies is a prevalent concern. As an example, they have already been successfully adapted and implemented for use in Nigeria, demonstrating their versatility in addressing the specific needs of various areas affected by canine rabies (https://anyadoc.shinyapps.io/ZeroRabiesNigeria/). These apps would be particularly useful in reducing the risk of rabies infection for travelers visiting dog rabies‐endemic countries.

Both the ZRA and ZRP applications are accessible via smartphones and computers, offering users flexibility and convenience. The smartphone penetration rate in India is close to 71% in 2023, and government agencies are already delivering public services via smartphones (https://www.statista.com/statistics/467163/forecast‐of‐smartphone‐users‐in‐india/ and https://www.india.gov.in/spotlight/mobile‐seva‐citizen‐services‐mobile‐phones#:~:text=M%2DGovernance%20aims%20to%20provide,public%20services%20through%20handheld%20devices). One limitation is that both ZRA and ZRP require an active internet connection to run. We are currently working on an Interactive Voice Response (IVR) to ensure access to point‐of‐care bite management guidelines as well as the rabies biologics database without an active internet connection.

Citizen scientists are playing an ever‐growing role in identifying and addressing local problems that benefit the public. The ZRA will be an invaluable resource for healthcare providers in canine rabies‐endemic regions, offering immediate access to the latest guidelines from the WHO and the Centers for Disease Control and Prevention (CDC). Additionally, community members such as pharmacists and hospital staff can use the app to guide illiterate patients, thereby enhancing compliance with PEP protocols. The use of precise, up‐to‐date, and freely accessible information to prevent infectious diseases like rabies empowers the communities most impacted at the local level. Indeed, when combined with large‐scale canine vaccination efforts and the provision of suitable human PEP, the utilization of real‐time databases, as demonstrated in these instances, constitutes an additional, highly effective measure for enhancing the response to rabies in regions where the disease is widespread. This approach aligns with the overarching principles of the One Health framework, benefiting both local residents and visitors alike.

## Author Contributions

Aniruddha V. Belsare developed the Shiny Apps. Aniruddha V. Belsare, Deborah J. Briggs, Gyanendra Gongal, Reeta S. Mani, and Charles E. Rupprecht made substantial contributions to the conception and design of the study and wrote the manuscript.

## Conflicts of Interest

The authors declare no conflicts of interest.

## Data Availability

Shiny App code will be made available upon request.
